# Appliances Therapy in Obstructive Sleep Apnoea: A Systematic Review and Meta-Analysis

**DOI:** 10.7759/cureus.48280

**Published:** 2023-11-04

**Authors:** Arushi Beri, Sweta G Pisulkar, Surekha A Dubey, Seema Sathe, Akansha Bansod, Akshay Shrivastava

**Affiliations:** 1 Prosthodontics, Sharad Pawar Dental College and Hospital, Acharya Vinoba Bhave Rural Hospital (AVBRH), Wardha, IND; 2 Prosthodontics and Crown & Bridge, Sharad Pawar Dental College and Hospital, Wardha, IND; 3 Prosthodontics, Sharad Pawar Dental College and Hospital, Wardha, IND; 4 Orthodontics and Dentofacial Orthopaedics, Kalinga Institute of Dental Sciences, Orissa, IND

**Keywords:** customized maxillary oral appliance, tongue retaining devices, soft palate lifters, sleep-disordered breathing, mandibular advancement therapy, oral appliance therapy, obstructive sleep apnea

## Abstract

Obstructive sleep apnea (OSA) is a recurrent partial or complete obstruction of the upper airway during sleep caused by narrowing or collapse of the pharyngeal wall. It leads to microstimulation and oxyhemoglobin desaturation, resulting in sleepiness and loud snoring. OSA negatively affects the cardiovascular system and may contribute to neurocognitive impairment. The aim of this systematic review is to evaluate the effectiveness and efficacy of appliance therapy in obstructive sleep apnea. The effectiveness was assessed by using the Apnea Hypopnea Index (AHI). An electronic search of the Cochrane Library, PubMed, and Google Scholar was conducted between 1998 and 2021. Articles were independently assessed by three reviewers. The quality of a randomised control trial (RCT) is assessed using the Cochrane risk of bias method. The tool GRADE was used to achieve the desired level of confidence for each outcome reported. Several studies used continuous positive airway pressure (CPAP), mandibular advancement devices (MAD), and tongue retention devices (TRD). The meta-analysis included a total of six papers that met the inclusion criteria. Results showed that CPAP significantly improved AHI compared with an oral appliance (random effects: difference in means = 8.40, 95% CI = 7.21 to 9.60). It was also found that oral appliance (OA) therapy significantly improved AHI compared with baseline before appliance therapy (random effects: mean difference = 13.40, 95% CI = 10.87 to 15.93; p.00001). For mild to moderate OSA, CPAP is considered the gold standard. Our meta-analysis of six RCTs found favorable evidence for OSA patients receiving oral devices; however, they were less effective than CPAP. A subgroup analysis found that MAD may be a beneficial treatment for mild to moderate OSA patients who do not respond to CPAP. The findings suggest that oral appliances may be an effective treatment for OSA, especially in patients with mild to moderate OSA.

## Introduction and background

Obstructive sleep apnoea (OSA) is a recurrent partial or complete obstruction of the upper airway during sleep caused by a narrowing or collapse of the pharyngeal wall. It leads to microstimulation and oxyhemoglobin saturation, resulting in fatigue and loud snoring [1,2]. OSA can damage the cardiovascular system and impair cognitive function [3]. Daytime sleepiness and behavioural disturbances have also been reported [4-7]. The risk of heart disease can be reduced by early detection and basic care in the form of treatments. It is undeniable that sleep apnea syndrome increases the risk of stroke and stroke-related death. After stroke, OSA can increase disability and stroke risk. The nocturnal sleep polygram is a diagnostic tool for OSA and is performed by a sleep physician [8]. Clinically, OSA presents as a soft ridge near the pharyngeal wall that does not allow the uvula to protrude, even when the voice is loud [9]. The purpose of this systematic review is to evaluate the effectiveness and efficacy of appliance therapy in obstructive sleep apnea. Dentists should ensure that these clinical studies allow for early diagnosis and appropriate treatment planning during the first appointment to avoid long-term sequelae. Consequently, dentists play an important role in the screening, diagnosis, and treatment of OSA patients. The Apnea Hypopnea Index (AHI) determines the severity of their OSA based on the number of apnea and hypopnea events per hour. Based on the AHI, OSA is classified as mild (AHI 5-15), moderate (AHI 15-30), or severe (AHI > 30) [10-13]. Physical therapy, medications, behavioural therapy, bariatric surgery, surgical procedures (pharyngeal and maxillofacial surgery), respiratory control (continuous positive airway pressure; CPAP), and oral appliances (OA) such as mandibular appliances (mandibular advancement devices; MAD) are used to treat OSA. [14]. Currently, over 60 different OAs are used in various models. However, it is not yet recognised as the gold standard. The customized maxillary oral appliance (CMOA) is a new medical device that targets the new OA and provides a new treatment option for OSA management. OA is used to treat OSA and is generally divided into three categories: soft palatal lift (SPL), mandibular advancement (MAS), and tongue restraining device (TRD) [15]. However, the modified OA showed the best results. Of these, MAD seems to be the most effective treatment. In MAD, the mandible moves forward and widens the upper airway. 

## Review

**Figure 1:**
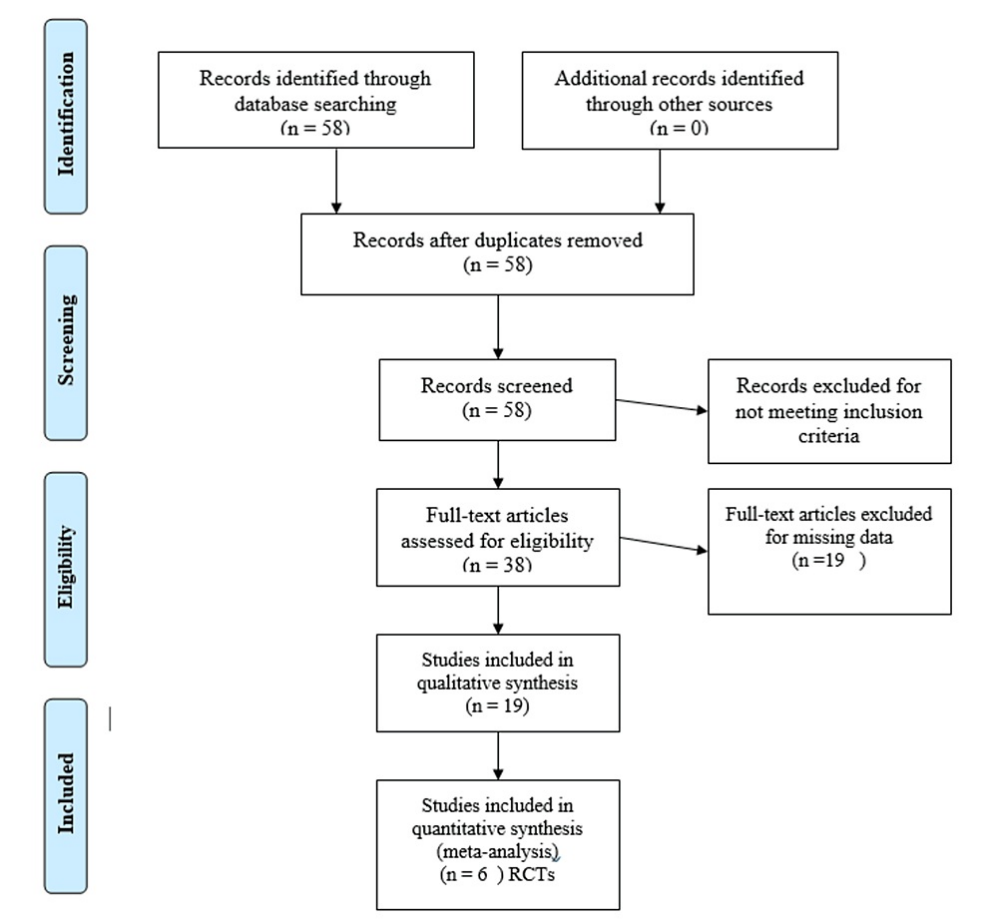
Flow chart for the selection of studies RCT, randomized controlled trial

Prospero registration was done with registration number CRD42022369150. We followed the Preferred Reporting Items for Systematic Reviews and Meta-Analyses (PRISMA) statement checklist in our systematic review [15]. Inclusion criteria using PICOS (population, intervention, comparison, outcome, and study design) was applied for the inclusion criteria. Population: patients diagnosed with apnea/hypopnea AHI >5 diagnosed with OSA, patients having CPAP; Intervention: mandibular advancement appliances, tongue retaining device; Comparison: CPAP; Outcome: primary outcome was the AHI, secondary outcomes included (1) oxygen saturation level and (2) ESS. Exclusion criteria include patients diagnosed with severe OSA, patients suffering from severe periodontal disease, an edentulous arch or a lack of teeth because there is insufficient retention for the appliance, patients suffering from temporomandibular joint diseases, airway blockage that is pathologically obvious; Data Sources/Searches: we began by searching MEDLINE, Google Scholar, and Scopus with various term and phrase combinations: "Obstructive Sleep Apnea Syndrome", "Apnea/Hypopnea Index", "AHI", "BMI", "Oral" Device, "Orthosis" "OUA", "Mandibular Advancement Therapy", "MAD", "MAA", "Soft Palate Elevator Device", "Long-Term Retention Device", "NCAP", "CPAP". All reviews and meta-analyses had their reference lists scoured. Data Extraction and Quality Assessment: two reviewers independently screened articles and abstracts. Each reviewer independently assessed the articles in detail found potentially relevant and obtained as per the said criteria. All discrepancies were resolved by consent. Figure 1 depicts the selection process flow chart. A total of 58 publications were found, 31 in PubMed, 21 in Scopus, and six in the Cochrane Library, with two more found through other sources. After reviewing the entire texts, 38 articles were judged to be of high reference value but did not match the inclusion requirements. Finally, the review contained 19 original research. The meta-analysis comprised six RCTs from these articles.

Keywords used for data extraction: “Obstructive sleep apnoea” “AHI,” “Mean AHI,” “oral appliances” “apnea/hypopnea index”, “MAD”.”NCAP” “CPAP” “Randomised”.”Randomization””Concealed”, “Blinding” CPAP, Customised, TRD, TSD, Search terms “Obstructive sleep apnoea” OR “Obstructive sleep apnoea syndrome” OR “Obstructive sleep apnoea hypopnoea syndrome” OR “OSA” OR “OSAH”, “apnea/hypopnea index”, OR “AHI,” “oral appliances in OSA,” OR “appliance therapy in OSA,” “mandibular advancement device OR“MAD”” ‘’NCAP” OR“CPAP” “MAD AND CPAP” “Oral appliances and CPAP” “Randomised controlled trial” OR “RCT” “Tongue retaining device” OR “tongue stabilizing device” OR “TRD” or “TSD” “[mh]” “[tiab]”. The rationale for selection of these keywords includes general information, eligibility, population, setting, methodology, risk of bias assessment, participants, intervention, comparison, outcome, result, and conclusion. Certainty level for the outcome AHI was achieved by using the GRADE instrument found to be moderate for the included studies. A summary of study characteristics and results of included studies is shown in Table 1.

**Table 1:** A summary of study characteristics and results of included studies AHI, apnea-hypopnea index; MAD, Mandibular advancement devices; TRD, Tongue restraining devices; ESS, Epworth sleepiness scale; EDS, excessive daytime sleepiness; OSA, Obstructive sleep apnea; SaO2, mean arterial oxygen saturation; FOSQ, Functional Outcomes of Sleep Questionnaire, ODI oxygen desaturation index; nCPAP, nasal Continuous Positive Airway Pressure

Reference	Year of Publication	Methods	Participants	Interventions	Outcomes
Aarab et al. [16]	2011	Parallel Randomised controlled trial	mild/moderate obstructive sleep apnea patients	MAD (n = 21) and nCPAP (n = 22)	AHI, events/h, EDS, Respiratory arousal
Schutz et al. [17]	2013	Parallel Randomised trial	25 OSA Patients	MAD (n = 9) and nCPAP (n = 9)	AHI, ESS
Phillips et al. [18]	2013	Randomized crossover trial	108 patients with moderate-severe OSA	MAD (n = 56) andnCPAP (n = 52)	AHI, OD, ESS
Nikolopoulou et al. [19]	2017	Randomised placebo-controlled trial	Participants were at least 18 years old with an AHI of 5–45 events per hour	MAD (n = 20) and nCPAP (n = 18). Placebo (n = 19)	AHI (events per hour), Total sleep time, Respiratory arousal index
Wojda et al. [20]	2018	Parallel randomised trial	16 OSA patients	MAD (n = 8) and nCPAP (n = 8)	AHI, ESS, SaO2
Banhiran et al. [21]	2019	Randomized crossover study.	Thirty-six patients with AHI ≥ 5 events/h	TRD (n = 13) and nCPAP (n = 14)	WatchPAT AHI, FOSQ, ESS, adverse side effects, and self-reported compliance

Risk of bias assessment

**Figure 2:**
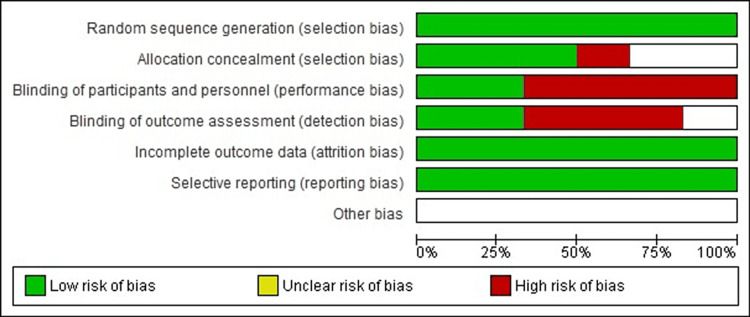
Risk of bias graph: Authors' judgments about all included studies. Risk of bias item presented in percentage.

The Cochrane Handbook of Systematic Review used RoB 1.0 (Risk of Bias 1.0) to assess seven domains such as sequence generation, allocation concealment, blinding of participants and personnel, blinding of outcome assessor, incomplete outcome, selective outcome reporting, and other bias if any. Each domain was assigned a risk of bias assessment of low, high, or unclear. Risk of bias is shown in Figure 2.

**Figure 3:**
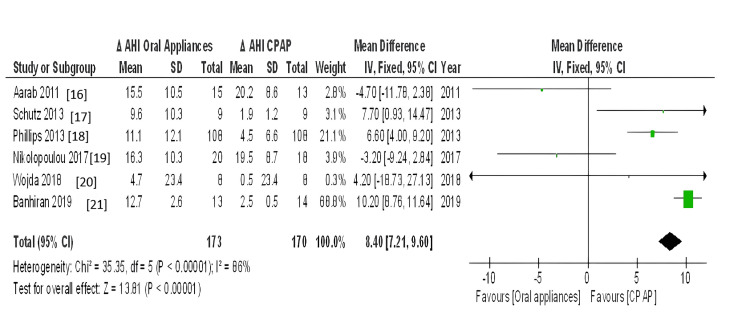
Forrest plot comparison of treatment effects (mean AHI) of oral appliances and CPAP. CI, confidence interval; df, degrees of freedom; IV, inverse variance; AHI, apnea-hypopnea index; ΔAHI, change in apnea-hypopnea index; SD, standard deviation, CPAP: Continuous Positive Airway Pressure

**Figure 4:**
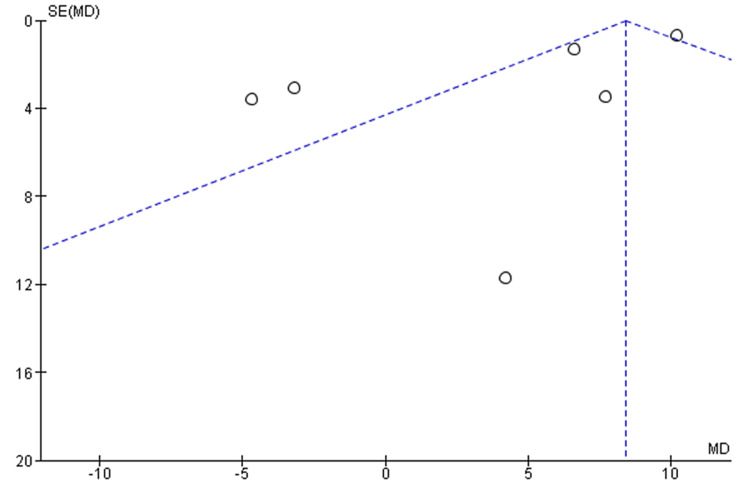
Funnel plot demonstrating mean AHI comparing the appliance therapy and CPAP AHI, apnea-hypopnea index; CPAP, continuous positive airway pressure

**Figure 5:**
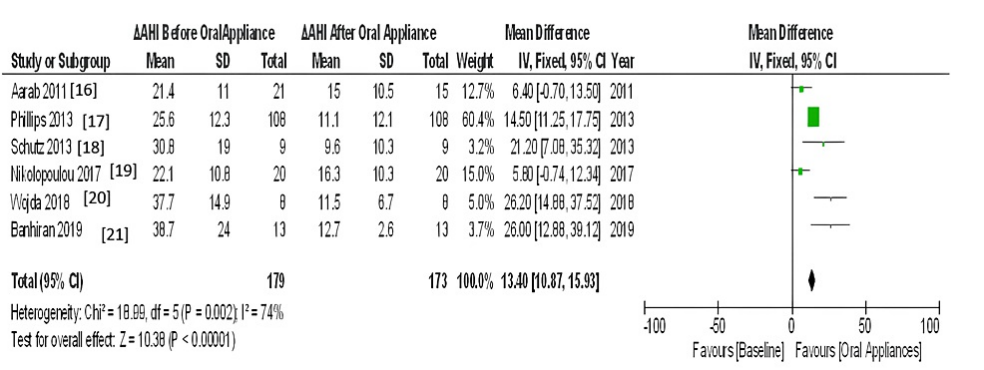
Plot of forest AHI before and after treatment with oral appliances was compared in six randomised controlled trials. CI, confidence interval; df, degree of freedom; IV, inversevariance; AHI, apnea-hypopnea index; SD, Standard deviation

**Figure 6:**
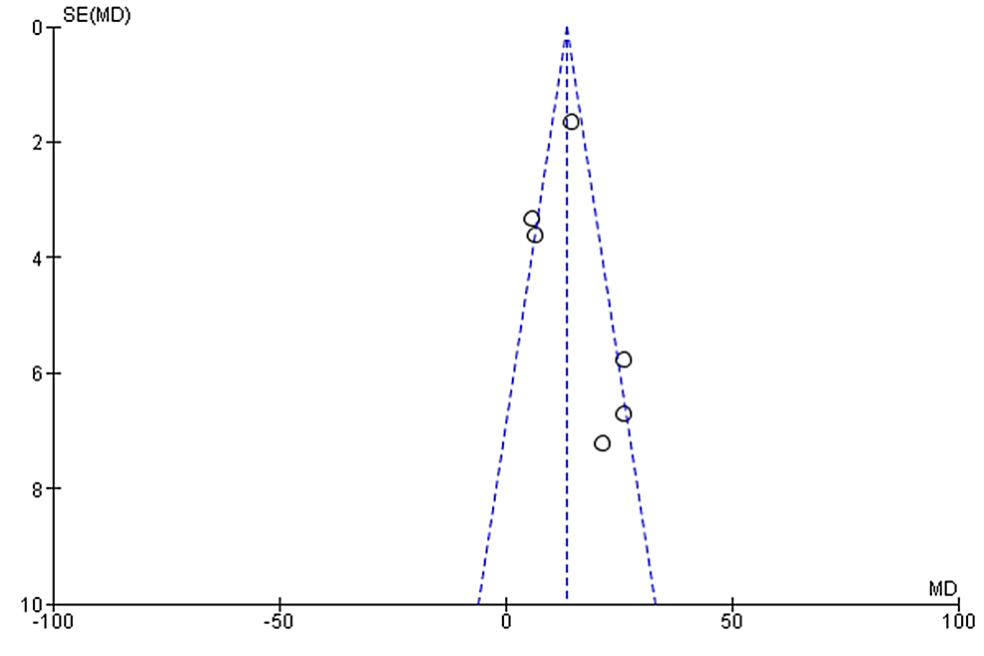
Funnel plot demonstrating mean AHI after the appliance therapy AHI, apnea-hypopnea index

The meta-analyses included only RCT studies on OSA patients comparing similar results with the same therapies (CPAP, MAD, and TRD). The effect estimate was the difference in means for all of the reported outcomes. When statistical heterogeneity (Q) (p value <0.10) was discovered, the random-effects model was utilised to report pooled findings. Sensitivity tests for the difference in outcome between baseline and post-treatment were performed using a different effect measure (standardised difference in means). For polysomnographic outcomes, we calculated the mean and standard deviation of AHI comparing oral appliance and CPAP patients, as well as before and after appliance therapy. Given the clinical and methodological variability of the available data, we pooled the incident rates and ORs using a random-effects model. RevMan5 was used for meta-analysis. Six trials reported the mean and standard deviation of AHI after therapy. Statistical heterogeneity was discovered (Q p <​​​​​​​.00001). I2=86% CPAP significantly reduced AHI compared to oral appliance users (random-effects: difference in means = 8.40, 95%CI=7.21 to 9.60; p <​​​​​​​0.00001). Figure 3 shows the forest plot comparison of treatment effects (mean AHI) of oral appliances and CPAP. Figure 4 shows the funnel plot demonstrating mean AHI comparing the appliance therapy and CPAP. Figure 5 shows the plot of forest AHI before and after treatment with oral appliances was compared in six randomized controlled trials. Figure 6 shows the funnel plot demonstrating mean AHI after the appliance therapy.

Three databases were scanned: MEDLINE, The Cochrane Library, and Scopus Manual search and review of studies to find the most relevant. The results of this study apply to all older men and women with mild to moderate OSA. The age of the participants ranged from 18 to 65 years. According to the results of this meta-analysis, many studies used CPAP, MAD, and TRD. The meta-analysis included six papers [16-21] that met the inclusion criteria. The results showed that CPAP significantly lowered AHI (negative effects: Mean difference = 8.40, 95% confidence interval = 7.21-9.60, p.00001) compared to oral appliances. The outcomes also revealed that oral therapy AHI (random effects; Mean difference = 13.40, 95% CI = 10.87-15.93. p.00001) compared to baseline before device treatment. Also, the outcomes revealed that CPAP improves its AHI more than an oral device. However, all studies have shown that oral devices significantly improve AHI compared to baseline and after oral device treatment compared to intravenous devices, and that oral devices significantly improve AHI compared to intravenous devices. Oral appliances could be an alternative to CPAP for OSA patients. Many other studies [22-24] have shown improvements in AHI in CPAP users compared to oral devices such as MADs. Although most studies have compared the oral device with mild CPAP in OSA, the use of MAD was as effective as CPAP in severe OSA with mandibular recession [25]. Our study compared oral appliances and CPAP in mild to moderate OSA, but studies comparing oral appliances and CPAP in severe OSA are needed. One study [26] showed that MAD and TRD had similar results in improving AHI. Studies [27-28] have shown that both CPAP and MAD show good long-term results when long-term treatment is initiated. The long-term efficacy of MAD and CPAP should be compared. Most studies involved the use of custom-made MADs. One study [29] showed that treatment with MAD was superior to his previous MAD and concluded that it had no effect on outcomes. Few studies [30-34] showed that this relationship may contribute to the development of AHI and MAD, representing the mandibular position relationship.

## Conclusions

OSA increases the risk of death considerably. The condition necessitates early multispecialty treatment. To guide focused therapy for OSA, new treatment regimen must be discovered, as well as streamlined phenotyping technologies for use in the clinic. Early evaluation and primary care in the form of OAs appears easily justified in controlling OSA in the community given the potential short- and long-term benefits as well as the negligible risk of the intervention. For mild to moderate OSA, CPAP is considered the gold standard. Our meta-analysis of six RCTs found favorable evidence for OSA patients receiving oral devices; however, they were less effective than CPAP. A subgroup analysis found that MAD may be a beneficial treatment for mild to moderate OSA patients who do not respond to CPAP.
